# Detection and Genotyping of Human Papillomavirus in Urine Samples from Unvaccinated Male and Female Adolescents in Italy

**DOI:** 10.1371/journal.pone.0079719

**Published:** 2013-11-08

**Authors:** Silvia Bianchi, Elena Rosanna Frati, Donatella Panatto, Marianna Martinelli, Daniela Amicizia, Carla Maria Zotti, Morena Martinese, Paolo Bonanni, Sara Boccalini, Rosa Cristina Coppola, Giuseppina Masia, Angelo Meloni, Paolo Castiglia, Andrea Piana, Roberto Gasparini, Elisabetta Tanzi

**Affiliations:** 1 Department of Biomedical Sciences for Health, University of Milan, Milan, Italy; 2 Department of Health Sciences, University of Genoa, Genoa, Italy; 3 Department of Public Health and Paediatrics Sciences, University of Turin, Turin, Italy; 4 Department of Health Sciences, University of Florence, Florence, Italy; 5 Department of Public Health, Clinical and Molecular Medicine, University of Cagliari, Cagliari, Italy; 6 Department of Biomedical Sciences, University of Sassari, Sassari, Italy; 7 Inter-University Centre for Research on Influenza and other Transmitted Infections (CIRI-IT), Department of Health Sciences, University of Genoa, Genoa, Italy; Alberta Provincial Laboratory for Public Health/ University of Alberta, Canada

## Abstract

The introduction of vaccination against Human Papillomavirus (HPV) in adolescent girls in 2006 has focused virological surveillance on this age group. As few studies have evaluated HPV infections in young populations, further data are needed in order to improve and extend prophylactic policy and to monitor epidemiological changes. The present study aimed at evaluating overall and type-specific HPV prevalence in both female and male adolescents in Italy. HPV DNA detection and genotyping was performed on urine samples collected from 870 unvaccinated adolescents (369 females, 501 males, 11-18 years of age) in five cities in Italy. Following DNA extraction by means of a commercial kit (NucliSENS^®^-miniMAG^®^, bioMérieux), the L1 gene fragment was PCR amplified and genotyped by restriction fragment length polymorphism analysis. HPV DNA was detected in 1.5% of all samples, and in 3% and 0.4% of samples from females and males, respectively. In approximately 70% of HPV DNA positive adolescents, the infection was due to a single genotype, with 88.9% of genotypes belonging to the HR-clade. The only two HPV-positive boys (14 and 18 years old) had HPV-70 genotype. Only one of the 11 HPV-infected girls was in the 11-14 age-group. HPV prevalence was 4.2% in girls aged 15-18 years and 60% of infections were due to vaccine types HPV-16 or HPV-6/-11. This is one of the few studies, the first conducted in Italy, on HPV infection in adolescents. Urine testing is the easier way of detecting HPV infection in younger populations. Our data revealed a very low HPV prevalence, and no infections were observed in the 12-year-old vaccine target population. The majority of infections were seen in females aged 15-18 years. Overall, more than 50% and 30% of the potentially persistent HPV infections detected in this group could have been prevented by the quadrivalent and the bivalent vaccines, respectively.

## Introduction

Human Papillomavirus (HPV) is the cause of the most common sexually transmitted infection and is associated with the development of severe diseases, including invasive cervical cancer, anogenital cancers and oral carcinomas, as well as benign manifestations (e.g. genital warts) [1].

HPV vaccination has been introduced as a preventive measure against cervical cancer, with young women as its primary target [2]. Epidemiological data collected worldwide have clearly shown that HPV infections are often acquired soon after the first sexual intercourse [3,4] and adolescents who are sexually active have particularly high rates of infection [5,6].

To date, two different vaccine formulations are able to protect against persistent infection caused by the most common oncogenic types (HPV-16 and -18) associated with the development of cervical cancer, one of which also protects against genotypes HPV-6 and -11, which are associated with genital warts [7]. These two HPV vaccines have been licensed by the U.S. Food and Drug Administration (FDA) in 2006 (the quadrivalent vaccine) and in 2009 (the bivalent vaccine) for use in females 9-26 years and 9-25 years of age, respectively [8,9].

The Italian Medicines Agency (AIFA) approved both vaccines in 2007 [10,11] and The Italian Superior Council on Health drew up a vaccination schedule aimed at achieving 95% vaccination coverage among 12-year-old girls over a five-year period [12,13]. The 12- year-old cohort is the ideal target population, since previous exposure to HPV is unlikely in this age group, the median age at sexual debut being 16 years among Italian females [14–16].

Noteworthy, recent evidence has shown that genital HPV infections in adolescent females can occur even before the first sexual intercourse, possibly owing to non-coital sexual behaviours [17,18], autoinoculation [19] or other putative non-sexual routes [20]. These observations underscore the importance of primary prevention through vaccination at a very young age.

The quadrivalent HPV vaccine was approved by the FDA and the European Medicines Agency (EMA) also for males 9-26 years of age in 2009 and in 2011, respectively [21,22]. The American Academy of Pediatrics (AAP) recommends HPV vaccination for boys, in order to obtain benefits both directly (protection against genital warts and anal or oral HPV-associated cancers) and indirectly (for females through herd immunity) [23]. However, there is no consensus regarding the advantages of male vaccination, and in many countries this vaccination strategy remains a point of debate [22].

Although adolescents are the primary target of prophylactic HPV vaccination programs, few epidemiological studies on this age group are available. Additional data are therefore needed in order to ascertain the HPV infection status of the adolescent population. This knowledge would enable us to improve and extend vaccination policy and to monitor changes in overall and type-specific HPV prevalence rates as a result of HPV vaccination. 

To add new epidemiological data to this issue, we conducted a study aimed at evaluating the prevalence of HPV infection and the distribution of HPV genotypes among unvaccinated 11- to 18-year-old boys and girls in Italy by means of a molecular test suitable for HPV DNA detection and genotyping in urine samples [24].

## Materials and Methods

### Ethics Statement

The study protocol used by all research units was approved by the Ethics Committee of the Genoa Local Healthcare Unit (LHU), Italy. All subjects gave assent to participate in the study. Informed written consent was obtained from each 18 year-old participant, or from parents or legal guardians if subjects were under 18 years of age. 

### Study population

As part of a National Research Program of the Italian Ministry of University and Research (MIUR), this study recruited HPV-unvaccinated male and female adolescents at sports medicine centres and at secondary schools located in five cities of five different Regions: Turin (Piedmont), Milan (Lombardy), Genoa (Liguria), Florence (Tuscany) in North/Central Italy, and Sassari (Sardinia), Insular Italy. On the basis of our preliminary studies (unpublished data), the expected prevalence for HPV positivity in urine from adolescents was around 1%, ranging from 0 to 3%. The power and the statistical significance (two sided) were set at 90% and 5% level, respectively. To determine the sample size in order to detect a difference between proportions in a range of 1% (i.e. between 0.5% and 1.5%), at least 865 individuals were needed to be enrolled. Individuals were proportionally sampled in the five cities on the basis of resident population of 11-18 years of age. Recruitment was conducted between September 2009 and July 2010 and each participant was asked to provide an anonymous self-collected urine sample. 

### Sample collection and pre-treatment

Urine samples were collected in sterile containers, maintained at room temperature (RT) and processed within 6-8 hours after collection at each participating centre. To obtain the cellular component, at least 15 ml of each sample was pre-treated by means of two different successive steps of centrifugation and washing, as previously described [24] and stored at -20°C until shipment to the centres of Milan (Lombardy) or Cagliari (Sardinia) for molecular analyses.

### DNA extraction and HPV DNA detection

HPV DNA was extracted from urine samples by means of the NucliSENS^®^ miniMAG^®^ (bioMérieux, Lyon, France) extraction kit. The concentration and purity of extracted DNA was evaluated by using a spectrophotometer (Thermo Scientific NanoDrop 2000; Thermo Fisher Scientific, Inc., Wilmington, DE, USA). DNA quality was assessed by amplifying a 268 bp (base pair) fragment of the ubiquitous β-globin gene. HPV DNA was detected through PCR amplification of a 450 bp segment of ORF L1 by using the degenerate primer pair ELSI-f and ELSI-r in previously reported amplification reaction conditions [24]. Each PCR run included positive controls (DNA extracted from HPV-16 positive cells, CaSki) and negative (water) controls.

The amplification products were visualized by means of electrophoresis analysis of 2% agarose gel containing ethidium bromide (0.5 mg/ml). Amplified product bands were compared with molecular weight standards (DNA Molecular Weight, Marker 100, Sigma-Aldrich, St. Louis, MO, USA). PCR products of approximately 450 bp were subjected to restriction fragment length polymorphism (RFLP) genotype analysis.

### Restriction Fragment Length Polymorphism (RFLP) analysis

Amplified products were digested with 1U of either RsaI, DdeI or HaeIII, diluted in their respective buffers and incubated at 37°C for 1 hour. Digestion products underwent electrophoresis in 3% agarose gels, and restriction patterns were compared with molecular weight standards (DNA Molecular Weight, Marker 100+20, Sigma-Aldrich, St. Louis, MO, USA). The pattern of fragments obtained from the three digestive processes enabled to genotype to be identified [25]. The RFLP analysis can identify all genotypes included in the high risk clade (HR-clade) and low-risk genotypes (LR) of the alpha genus according to the latest IARC classification system [1].

### Statistical analysis

Sample size was calculated using the statistical software Stata 11.0 (StataCorp LP, College Station, Texas). HPV prevalence was expressed as a crude proportion with corresponding 95% confidence intervals (95% CI) calculated using the Mid-p exact test assuming a normal distribution. A P-value <0.05 was considered statistically significant (2-tailed test). Statistical analysis was performed using OpenEpi, version 3.01 [26].

## Results

### Demographic characteristics of the study population

A total of 870 adolescents aged 11-18 years (median age 15.5 years; IQR 14-17) were enrolled: 501 males (median age 16.0 years; IQR 14-17) and 369 females (median age 15.0 years; IQR 14-17). The final proportions of recruited individuals in the five cities did not significantly differ from those expected by sample calculation (Chi squared P = 0.35): 34.0% of urine samples were collected from adolescents in Turin, 30.7% in Milan, 21.4% in Genoa, and 8.6% and 5.3% from boys and girls in Florence and Sassari, respectively. The median age and the male/female distribution of the participants was comparable between the cities involved.

### HPV detection and genotyping

The β-globin gene was amplified from all 870 urine samples collected, confirming the suitability of the extraction protocol. Overall, 13 (1.5%; 95%CI 0.8-2.5) subjects were HPV DNA-positive (11 females and 2 males).

The geographic distribution of HPV-positive adolescents was as follows: 0.7% (2/296; 95%CI 0.1-2.2) in Turin, 1.5% (4/267; 95%CI 0.5-3.6) in Milan, 2.7% (5/186; 95%CI 1.0-5.9) in Genoa, and 4.3% (2/46; 95%CI 0.7-13.6) in Sassari. HPV DNA was not detected in samples collected from participants in Florence (0/75; 95%CI 0.0-3.9). No significant differences were observed.

Overall, 17 different infections were identified. In 69.2% (9/13; 95%CI 41.3-89.4) of HPV DNA positive adolescents, the infection was due to a single genotype and 88.9% (8/9; 95%CI 56.1-99.4) of the genotypes identified belonged to the HR-clade. The remaining 4 subjects (30.8%; 95%CI 10.6-58.7) had double infections, two of which were sustained by LR HPV types and two by at least one genotype belonging to the HR-clade.

HPV prevalence rates were significantly different between genders (P<0.05): 0.4% (2/501; 95%CI 0.1-1.3) and 3.0% (11/369; 95%CI 1.6-5.1) in males and females, respectively (Table 1). 

**Table 1:** Prevalence of positive subjects, broken down by gender and age-group (11-14 and 15-18 year-olds).

	**Males**		**Females**		**Population Enrolled**	
	**HPV-positive**	**95% CI**	**HPV-positive**	**95% CI**	**HPV-positive**	**95% CI**
	**subjects N (%)**		**subjects N (%)**		**subjects N (%)**	
11-14 years	1/162 (0.6)	0.0-3.0	1/132 (0.8)	0.0-3.7	2/294 (0.7)	0.1-2.2
15-18 years	1/339 (0.3)	0.0-1.5	10/237 (4.2)	2.2-7.4	11/576 (1.9)	1.0-3.3
**Total**	2/501 (0.4)	0.1-1.3	11/369 (3.0)	1.6-5.1	13/870 (1.5)	0.8-2.5

Among the 11 HPV DNA-positive girls (mean age 16.5 years, range 14-18 years), only one (14 years old) belonged to the 11-14-year age-group (1/132, 0.8%; 95%CI 0.0-3.7). In the female 15-18 age-group, the prevalence of HPV infection was 4.2% (10/237, 95%CI 2.2-7.4), significantly higher than that detected in 11-14 age-group (P<0.05) (Table 1).

In the two HPV DNA-positive boys (14 and 18 years of age) the infection was attributed to HPV-70. Approximately sixty-four percent (63.6%; 95%CI 33.6-87.2) of the 11 HPV-positive girls had a single infection and 4 (36.4%; 95%CI 12.8-66.4) were infected with two genotypes. A total of 8 different genotypes were detected among 15 infections identified in females: HPV-16 (4/15, 26.7%; 95%CI 9.1-52.5), HPV-70 (3/15, 20.0%; 95%CI 5.3-45.4), HPV-6 and HPV-11 (2/15 each, 13.3%; 95%CI 2.3-37.5) and HPV-52, HPV-54, HPV-66 and HPV-87 (1/15 each, 6.7%; 95%CI 0.3-28.7) (Table 2). 

**Table 2:** HPV infections and HPV infecting types in females, broken down by age-group.

	**11-14 age-group N (%**)	**95% CI**	**15-18 age-group N (%**)	**95% CI**
**HPV INFECTIONS**	1 (9.1)	0.5-37.3	10 (90.9)	62.7-99.6
Single infections	1 (9.1)	0.5-37.3	6 (54.5)	25.9-81.0
Multiple infections	-	-	4 (36.4)	12.8-66.4
**HPV INFECTING**	1 (6.7)	0.3-28.7	14 (93.3)	71.3-99.7
TYPES*				
HR group 1	-	-	5 (33.3)	13.4-59.2
			HPV-16: 4 (26.7)	
			HPV-52: 1 (6.7)	
HR group 2A/2B	1 (6.7)	0.3-28.7	3 (20)	5.4-45.4
	HPV-70: 1 (6.7)		HPV-70: 2 (13.3)	
			HPV-66: 1 (6.7)	
Group 3/LR	-	-	6 (40)	18.1-65.5
			HPV-6: 2 (13.3)	
			HPV-11: 2 (13.3)	
			HPV-54: 1 (6.7)	
			HPV-87: 1 (6.7)	

* According to the new IARC classification system [19]: Group 1 = carcinogenic to humans; Group 2A (HPV-68) = probably carcinogenic to humans; Group 2B = possibly carcinogenic to humans; Group 3/LR = not classifiable as carcinogenic to humans.

The only HPV positive girl among the 11-14-year age-group was infected with HPV-70. Sixty per cent of the positive girls in the 15-18 age-group were infected with the HPV-16 vaccine type or co-infected with the LR- types HPV-6 and -11 included in one of the two available vaccines.

## Discussion

To our knowledge, no research has been planned with the aim to evaluate HPV prevalence rates and genotypes distribution in adolescents (11-18 year-old) in Italy prior to our study. An extensive survey carried out in Italy on the prevalence of high-risk HPV infection in women aged 15-73 years found a high prevalence (29.5%) in females between the ages of 15-19 years, but the number of participants was very low and they were all sexually active [27].

Worldwide, several studies have analysed adolescents within a broader age range and by means of different methods and types of sampling, making the results difficult to compare [28]. Only two studies have evaluated HPV infections in urine samples collected exclusively from adolescents [29,30].

The present study identified a very low prevalence rate (1.5%) of HPV infection among Italian adolescents, and found a significantly lower prevalence in boys (0.4%) than in girls (3.0%) (P<0.05). Our findings are consistent with those reported in India by Hussain et al. [30] that showed a low HPV prevalence rate in females and males between the ages of 8 and 17 years (3.2% and 2.1%, respectively), which increased with age. In contrast, O'Leary et al [29] reported prevalence rates of 1.1% and 1.4% in 11-14-year-old females and males respectively, and of 15.2% and 3.9%, respectively, among those aged 15-18 years in Scotland. The differences observed could be explained by socio-economic, cultural, and geographical differences, as well as by differences in the modes of recruitment and methodologies used to establish HPV infections. 

In the present study, no significant differences were observed in HPV infection prevalence rates among adolescents residing in the five cities involved. A slightly, but not significantly, higher percentage of infection was noted among adolescents from Sassari. This may have been due to the low number of samples analysed. However, the Sassari area is known to have a high prevalence of HPV infections, since high rates have been reported in women aged 15-54 years (35.9%) and 15-24 years (53.9%) undergoing Pap-testing at public and private outpatient clinics [31].

In the 11-14-year age-group, only one female, aged 14 years, proved HPV-positive (1/132, 0.8%); this infection was not associated with the vaccine types. No infections were observed in the primary target vaccination group (12-year-old females).

The majority (57%) of infections found in the 15-18-year-old females were caused by genotypes belonging to the HR-clade, with HPV-16 in 50% of cases. Overall, more than 50% and 30% of the potentially persistent HPV infections detected in this group could have been prevented by the quadrivalent and the bivalent vaccines, respectively. 

The only two HPV-positive males were infected with HPV-70. This genotype, that is classified by the IARC as "possibly carcinogenic to humans" (group 2B), is not infrequently detected in adolescents. Indeed, it has been shown to be prevalent among males aged 15-18 years in Scotland [29]. Furthermore HPV-70 was responsible for 27% of infections in the young females examined in our study. A more accurate monitoring of the circulation of this genotype could better determine its potential oncogenic role.

The method used in the present study appears to be adequately able to identify HPV infections in urine samples, since the pre-treatment required [24] allowed the detection of genomic DNA that was valid for molecular testing in all samples.

Several studies have validated urine sampling as an alternative to cervical sampling-based methods for detecting HPV infections in women [24,28,32,33]. Moreover, the urine collection seems to be the best solution for young girls, being non-invasive and more acceptable, bypassing the medical examination and ethical issues. For these reasons this approach can be used to carry out virological surveillance in female adolescents in order to assess changes in HPV prevalence rates and to determine genotypes circulation after the introduction of prophylactic vaccination.

In males, the analysis of urine samples for the detection of HPV remains controversial, since there is no ‘gold standard’ sampling method, as there is in females (i.e. analysis of cervical swabs), and few studies have compared the sensitivity of HPV detection in urine with that of detection in other urogenital samples (e.g. penile/urethral swabs). Furthermore, no studies have compared this detection method with other techniques in male adolescents. Therefore, a consensus regarding which sampling method in males is most effective is still lacking [34,35].

Although the data presented in this report support the adequacy of urine sampling for the diagnosis of HPV infections in males, the prevalence of HPV infections reported may be underestimated owing to low viral DNA concentrations in urine or to presence of HPV infections in anatomical sites not covered by urine transit [28,35].

A further limitation of the study was the lack of information on the socio-demographic characteristics, sexual debut and sexual habits of the subjects studied. However, this was not the primary objective of the study, which was designed to evaluate the overall and type-specific HPV prevalence in an unvaccinated adolescent population in Italy.

## Conclusions

This is one of few studies, and the first conducted in Italy, designed to establish HPV prevalence and genotypes distribution in an adolescent population. The data reported suggest a very low HPV prevalence rate in the age-group studied. The fact that no infections were observed in the primary target vaccination group (12-year-old females) indicates that the prevention strategy currently in force in Italy is appropriate. Furthermore, the low prevalence of HPV infections observed in 15-18-year-old females supports the multi-cohort strategy of vaccination adopted by several Italian Regions [36], also considering that more than 50% and 30% of the potentially persistent infections detected in this age-group could have been prevented by the quadrivalent and bivalent formulations, respectively. 

Molecular testing of urine samples is the non-invasive and easier way to determine the HPV infection status in the youngest age-groups. This is essential to establishing the baseline HPV prevalence, which is a prerequisite to monitoring the impact of HPV vaccination at the population level. Future studies will have to be conducted on a larger population of adolescents representing different geographical areas and socio-cultural features in order to provide a more accurate estimate of HPV infection status in this age group.
